# Functional Effects of Hyperthyroidism on Cardiac Papillary Muscle in Rats

**DOI:** 10.5935/abc.20160179

**Published:** 2016-12

**Authors:** Fabricio Furtado Vieira, Robson Ruiz Olivoto, Priscyla Oliveira da Silva, Julio Cesar Francisco, Rosalvo Tadeu Hochmuller Fogaça

**Affiliations:** 1 Laboratório de Fisiologia da Contração Muscular - Universidade Federal do Paraná - Brazil; 2 Instituto de Pesquisa Pelé Pequeno Príncipe, Curitiba, PR - Brazil

**Keywords:** Hyperthyroidism / metabolism, Rats, Myocardium, Myocardial Contraction, Thyroid Hormones

## Abstract

**Background:**

Hyperthyroidism is currently recognized to affect the cardiovascular system, leading to a series of molecular and functional changes. However, little is known about the functional influence of hyperthyroidism in the regulation of cytoplasmic calcium and on the sodium/calcium exchanger (NCX) in the cardiac muscle.

**Objectives:**

To evaluate the functional changes in papillary muscles isolated from animals with induced hyperthyroidism.

**Methods:**

We divided 36 Wistar rats into a group of controls and another of animals with hyperthyroidism induced by intraperitoneal T3 injection. We measured in the animals' papillary muscles the maximum contraction force, speed of contraction (+df/dt) and relaxation (-df/dt), contraction and relaxation time, contraction force at different concentrations of extracellular sodium, post-rest potentiation (PRP), and contraction force induced by caffeine.

**Results:**

In hyperthyroid animals, we observed decreased PRP at all rest times (p < 0.05), increased +df/dt and -df/dt (p < 0.001), low positive inotropic response to decreased concentration of extracellular sodium (p < 0.001), reduction of the maximum force in caffeine-induced contraction (p < 0.003), and decreased total contraction time (p < 0.001). The maximal contraction force did not differ significantly between groups (p = 0.973).

**Conclusion:**

We hypothesize that the changes observed are likely due to a decrease in calcium content in the sarcoplasmic reticulum, caused by calcium leakage, decreased expression of NCX, and increased expression of a-MHC and SERCA2.

## Introduction

A normal endocrine function is essential for the cardiovascular health.^1^ Hyperthyroidism is among the most common endocrine disorders, with a prevalence of 1.3% in the United States^2^ and 0.7% (95% confidence interval [CI] 0.2-1.1%) in Brazil.^3^ This condition is defined by increased levels of thyroid hormones (T3 and/or T4) and suppressed or decreased levels of TSH.^2^

It is now recognized that thyroid hormones affect the cardiovascular system. Changes in these hormones' circulating levels influence the cardiac contractility and electrophysiological function.^4^ Increased thyroid hormone levels (hyperthyroidism) result in increased cardiac contractility, speed of contraction and relaxation, cardiac output, and heart rate.^1,5^

Thyroid hormones regulate a variety of proteins in the cardiac myocyte (including myosin heavy chains [MHC] a and β, β-adrenergic receptors, SERCA2, and phospholamban [PLB]) and may lead to cardiac hypertrophy.^6-8^ These molecular changes ultimately affect the cellular calcium cycling.^9^

Due to the great importance of calcium as a signaling pathway in the generation of membrane depolarization, induction of calcium release by the sarcoplasmic reticulum (SR), and activation of the contractile machinery, pathophysiological conditions that alter the control of calcium in the myocyte are the main causes of contractile dysfunction in the cardiac muscle and development of arrhythmias.^10^

However, little is known about the thyroid hormones influence on cellular events associated with increased and decreased cytoplasmic calcium in the excitation-contraction coupling process in the cardiac muscle.^11^ The largest amount of information available comprises changes in gene expression of proteins involved in the excitation-contraction coupling. Still, the functional consequences of these proteins in hyperthyroidism are still largely unknown. Much less is known about the consequences of changes in the sodium/calcium exchanger (NCX) on the cardiac muscle function in hyperthyroidism.

The objective of this study was to evaluate the effects of hyperthyroidism on the cardiac function, including cardiac strength and contraction time and speed in papillary muscles isolated from rats.

## Methods

The study included 36 male Wistar rats weighing 250-300 g, provided by the Animal Facility of the Department of Biological Sciences at *Federal University of Paraná* (UFPR). All animals were kept in cages under controlled temperature and 12-hour light-dark cycles, with free access to food and water. All procedures performed in this study were approved by the Ethics Committee on Animal Use (CEUA) at the Department of Biological Science, *Federal University of Paraná* (UFPR); Certificate No. 23075.098041/2011-11.

The animals were randomly divided into two groups: control (n = 18) and hyperthyroid (n = 18). Hyperthyroidism was induced by daily intraperitoneal injections of T3 (15 µg/100 g) during 10 days. The control group received daily injections of saline solution during the same period.^12,13^

After 10 days of treatment, the animals were anesthetized with ketamine (50 mg/kg) and xylazine (20 mg/kg), and subjected to thoracotomy. Their hearts were removed and placed in Ringer solution (pH = 7.4; 110 mM NaCl, 4 mM KCl, 2 mM CaCl_2_, 2 mM MgCl_2_, 10 mM TRIS, 11 mM glucose). The hearts were subsequently weighed and fixed in a petri dish containing oxygenated Ringer solution prior to dissection of the left ventricle papillary muscles.

After dissection and removal of the papillary muscles, one end of the muscle was fixed to a micromanipulator and the other was attached to a force transducer (Fort 10 WPI, Transduction Laboratories Co.), which, in turn, was connected to a data acquisition system (LabChart - ADinstruments). The muscle was stretched to the length at which the active voltage was maximal (Lmax) and kept in a chamber with a continuously oxygenated saline solution at 32ºC.

Before each experiment, a calibration curve was obtained for the force transducer using known masses. The length of the papillary muscle was measured using a microscope eyepiece graticule. At the end of each experiment, the papillary muscles were weighed. The area of the transverse section was calculated using the formula: area = mass / (length x density), assuming the density as 1.0. Thus, the strength produced by the papillary muscles was normalized by their cross-sectional area. After these steps, the experimental protocol was initiated.

To evaluate the effects of the thyroid hormone administration, the animals in both groups were exposed to two experimental protocols. The first protocol involved several contractility measurements of isolated and electrically stimulated papillary muscles. The second protocol evaluated the contraction force induced by caffeine in quiescent papillary muscles (not electrically stimulated).

### Experiments with electrically stimulated papillary muscle

After the papillary muscles were isolated and attached to a force transducer, as described above, the muscles were electrically stimulated with suprathreshold voltage pulses (10 to 15 V), with a maximum duration of 5 milliseconds, using a pair of platinum electrodes positioned along the entire muscle length. Using a micromanipulator, the muscle was then stretched to the Lmax. The standard stimulation frequency was 0.5 Hz (stabilized condition). The preparations were maintained in this condition during a stabilization period of 20-30 minutes and the experimental protocols were then conducted. The resultant force was recorded by a data acquisition system (LabChart - ADinstruments) connected to a computer.

The following contractile parameters were analyzed: maximum isometric force developed; post-rest potentiation (PRP), which is the increase in isometric contraction force obtained after electric stimulation pauses of 1, 3, 5, 10, and 20 seconds; maximum speed of contraction (+df/dt) and relaxation (-df/dt); total contraction time; time to peak contraction; time to maximum relaxation; and maximum contraction force at different concentrations of extracellular sodium.

For the PRP protocol, the electrical stimulation of the papillary muscles was interrupted after periods of stabilization of 1, 3, 5, 10, and 20 seconds. The amplitude of the first contraction after rest was compared with the amplitude of the force obtained before the resting period. These data were analyzed and expressed as percentage values of force obtained in the steady state prior to the resting period.

After stabilization in Ringer solution containing 110 mm NaCl, the preparation was placed in chambers with Ringer solution containing 90, 70, and 50 mM of NaCl. To maintain their osmolarity and ionic strength, the solutions were supplemented with lithium chloride (LiCl) at concentrations of up to 110 mM. The contraction amplitude was analyzed and expressed as percentage relative to the force value obtained in the solution with 110 mM of NaCl.

Both +df/dt and -df/dt were calculated in real time using the LabChart software. Data are expressed as force produced per cross-sectional area of muscle per second (mN/mm^2^/sec). Time to 100% of maximum force and time to maximum contraction force until 100% relaxation are expressed in seconds.

### Evaluation of the contraction in quiescent papillary muscles

Dissection and assembly of the papillary muscles in this experiment followed the same protocols described above, with the exception of the electrical stimulation.

This protocol was then carried out in three chambers containing different solutions. The first chamber contained Ringer solution. The second contained Ringer solution without sodium or calcium (Ringer 0Na^+^ - 0Ca^2+^), in which the sodium and calcium ions were replaced by lithium chloride in order to maintain the osmolarity and ionic strength equal to those in the Ringer solution. The third chamber was filled with Ringer solution 0Na^+^ - 0Ca^2+^ plus 30 mM of caffeine. According to the literature, caffeine is a known agonist of ryanodine receptors, and induces calcium release from the SR at concentrations of 30 mM.^14^

The preparation was initially immersed in Ringer solution for at least 30 minutes. It was then transferred to the Ringer solution 0Na^+^ 0Ca^2+^ and maintained for enough time to allow the force to reach the steady state (usually between 5 to 10 minutes). Then the papillary muscles were transferred to the chamber containing Ringer 0Na^+^ - 0Ca^2+^ plus 30 mM caffeine. The contraction force induced by caffeine was then compared between groups.

### Statistical analysis

The results were collected from at least six observations from each experiment and are expressed as mean ± standard error. The normality of the data was analyzed with the Shapiro-Wilk test, and the data were compared using Student's *t* test for independent samples. Statistical differences among groups were considered when p < 0.05. The software SigmaPlot (version 11.0) was used for data analysis.

## Results

Table 1 shows the body weight values on treatment days 1 and 10 and the heart weight values in both groups. After 10 days of treatment with the thyroid hormone, the animals in the hyperthyroid group showed a statistically significant decrease in body weight (p = 0.034) and increase in heart weight (p < 0.001) when compared with the animals in the control group.

**Table 1:** Animals’ weights on the first and tenth days after treatment

x00A0;	n	Animals’ weights (g) on the 1st day	Animals’ weights (g) on the 10th day	Heart weight (g)
Control	18	311 ± 10.94	336.2 ± 7.32	1.528 ± 0.036
Hyperthyroid	18	310 ± 9.57	309.7 ± 9.51 *	2.153 ± 0.074 ^#^

* p = 0.034;

# p < 0.001

**Figure 1:**
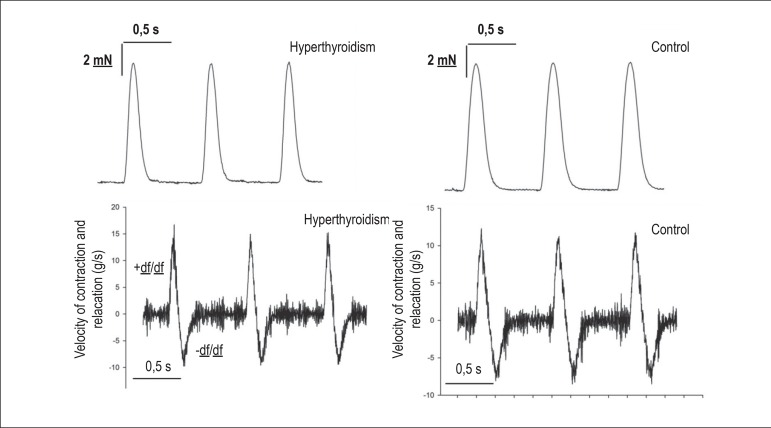
Maximum speed of contraction (+df/dt) and relaxation (-df/dt). The hyperthyroid group (n = 18) showed an increase in +df/dt and -df/dt when compared with the control group (n = 18).

The results of the maximum isometric contraction force in electrically stimulated papillary muscles, +df/dt, -df/dt, and total contraction time are shown in Table 2 and Figure 1.

**Table 2:** Force, speed, and contraction time parameters (n = 36)

x00A0;	Control	Hyperthyroid	p Value
Maximum force of isometric contraction (mN/mm2)	4.903 ± 0.13	4.917 ± 0.35	0.973
+df/dt (mN/mm^2^/s)	69.88 ± 2.77	105.90 ± 7.31	<0.001
-df/dt (mN/mm^2^/s)	51.92 ± 2.04	67.32 ± 3.59	<0.001
Total time of contraction (s)	0.441 ± 0.00	0.350 ± 0.00	<0.001
Time to peak contraction (s)	0.138 ± 0.01	0.108 ± 0.01	<0.001
Time to maximum relaxation (s)	0.303 ± 0.01	0.241 ± 0.01	<0.001

**Figure 2:**
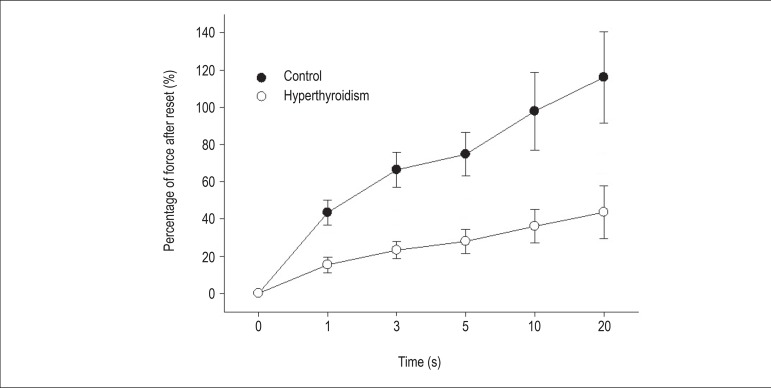
Percentage of strength after rest. The force gain at all resting times was significantly lower in the hyperthyroid group (n = 18) compared with the control group (n = 18) (*p < 0.001, # p < 0.05).

**Figure 3:**
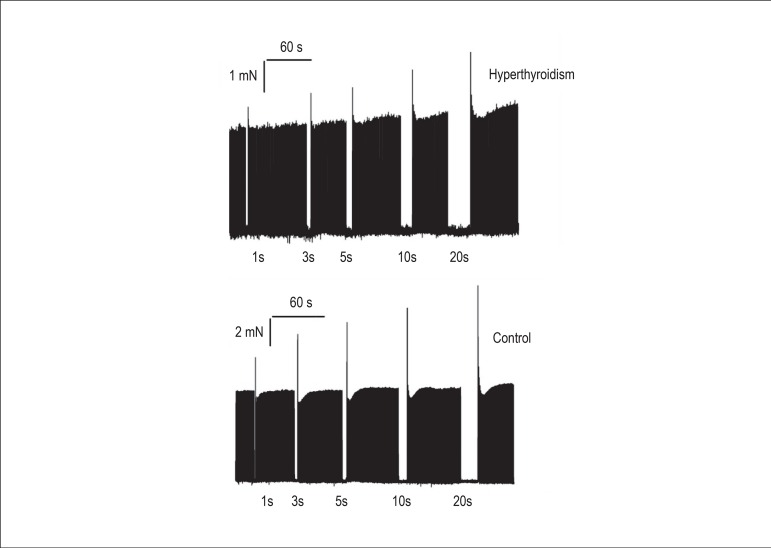
Post-rest potentiation (PRP). The increase in isometric contraction force after electrical stimulation pauses of 1, 3, 5, 10, and 20 seconds was lower in the hyperthyroid group (n = 18) compared with the control group (n = 18).

Regarding the PRP, the hyperthyroid group had a significant reduction in the percentage of strength gain at all pause times when compared with the control group (Figures 2 and 3).

**Figure 4:**
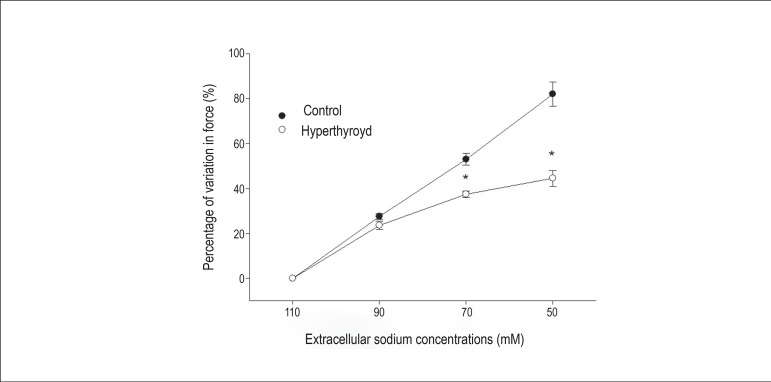
Percentage of force variation for different extracellular sodium concentrations. The force gain at different extracellular sodium concentrations was significantly lower in the hyperthyroid group in two conditions: extracellular sodium concentrations of 70 and 50 mM (n = 36, *p < 0.001).

Variations in the percentages of force generated at different extracellular sodium concentrations are expressed with reference to the force produced in a solution with 110 mM of extracellular sodium. A significant difference between groups was detected only at extracellular sodium concentrations of 70 mM (p < 0.001) and 50 mM (p < 0.001). At both times, the percentage of force gain in the hyperthyroid group was lower than that in the control group (Figure 4).

Regarding the maximum contraction force (mN/mm^2^) induced by caffeine in quiescent papillary muscle, we found statistically significant differences between the groups (p < 0.001). The contraction force in the hyperthyroid group was lower than that in the control group (3.26 ± 0.88 mN/mm^2^ versus 8.13 ± 1.07 mN/mm^2^, respectively).

## Discussion

In this study, we found that papillary muscles isolated from hyperthyroid rats showed decreased PRP, increased +df/dt and -df/dt, reduced contraction and relaxation total times, reduced maximal force induced by caffeine, and low positive inotropic response to decreased concentration of extracellular sodium. However, no difference in the isometric contraction force was observed.

The increase in +df/dt and the reduction in the contraction time may be explained by increased a-MHC expression.^6-7,15,16^ Although evidence is limited, an increased expression and function of ryanodine receptors, SERCA2, and L-type calcium channels may have contributed to these results.^11,17,18^ These changes lead to increased calcium influx, rate of calcium release by ryanodine receptors, speed reuptake of calcium by SERCA2, and a-MHC ATPase activity, which may explain the increase in +df/dt, ultimately leading to a decreased time to reach the contraction peak.^15,19,20^

The SERCA2 to PLB ratio is an important determinant in calcium uptake kinetics in cardiac myocytes, influencing both the relaxation rate and force production.^21^ Hyperthyroid animals have shown an increased SERCA2/PLB ratio, due to a decreased amount of PLB and increased SERCA2.^7,21-24^ Increases in the amount of phosphorylated compared with non-phosphorylated PLB have also been reported.^7^ Altogether, these changes promote increases in the calcium uptake rate by the SR, causing increased relaxation speed of the cardiac muscle (-df/dt) and, consequently, decreased relaxation time.^21,24^

In the myocardium of most mammals, PRP is believed to be produced by the release of a larger amount of calcium stored in the SR during the pause period.^25,26^ However, a loss of cellular calcium during rest is well described in the ventricles of rabbits, cats, and guinea pigs. These animals, therefore, lack PRP, especially over long periods of rest. However, PRP occurs in mice and is accompanied by increased calcium stored in the SR.^26^

In this study, papillary muscles isolated from hyperthyroid animals showed both a reduction in the PRP and maximal force induced by caffeine, an agonist of the ryanodine receptor. The concentration of caffeine used in this study (30 mM) is sufficient to deplete the calcium from the SR. These data suggest that hyperthyroidism reduces the amount of calcium stored in the SR.

This decrease in calcium content in the SR may be due to three not mutually excluding possibilities: a) an increase in NCX expression and/or activity, which in the resting stimulation period would increase the extrusion of calcium from the cell, decreasing the amount of calcium available for uptake by the SR; b) decreased SERCA2 expression and/or activity, and c) increased calcium leakage from the SR through the ryanodine receptors.^27^

Our results on contractility, along with data from the literature, lead us to hypothesize that the SERCA2 function is increased and the NCX function is decreased. However, these two changes are unable to reduce the PRP, so we believe that this occurred due to the release of calcium by the SR ryanodine receptors.

Another factor that could have contributed to the decreased calcium content in the SR is the phosphorylation of the ryanodine receptors by calcium/calmodulin-dependent protein kinase II (CaMKII). Studies have shown that hypertrophic conditions and heart failure, both common in hyperthyroidism, increase the expression of CaMKII, favoring the opening of ryanodine receptors that lead to leakage of calcium from the SR.^28^

Ai et al.^27^ found increased levels of CaMKII and phosphorylation of ryanodine receptors in animals with heart failure, strengthening the hypothesis of the receptors opening and favoring diastolic calcium leakage from the SR.^29,30^

Song et al.^31^ induced cardiac hypertrophy with thyroid hormone in rats and observed a decrease in the content of calcium in the SR due to increased calcium leakage by ryanodine receptors. They observed in animals that developed hypertrophy that the reduction in the release of calcium during contraction could be due to increased spontaneous calcium release at rest.

 Therefore, phosphorylation of ryanodine receptors by CaMKII increasing the spontaneous diastolic release of calcium from the SR could contribute to the decreased calcium in the SR.^27,28^ However, our model of hypertrophy was induced by thyroid hormone, and we were unable to find studies evaluating the expression of CaMKII in this model of hypertrophy.

The reduction in extracellular sodium concentration induces a positive inotropic response. Decreases in the electrochemical gradient of sodium reduce or even revert the NCX function, leading to increased intracellular concentration of calcium and, consequently, increased force.^28,32,33^

Diedrichs et al.^33^ have shown that the cardiac muscle in patients with heart failure is more sensitive to a reduction in extracellular sodium, exhibiting a greater increase in contractile force when compared with the muscle in normal individuals. According to the authors, this is due to intracellular calcium accumulation via NCX, whose expression and function is increased in heart failure.

Our results show that the contraction force in hyperthyroid animals was slightly increased when compared with that in controls. This may be explained by a reduced NCX function in hyperthyroidism. It is well described that hyperthyroid animals have decreased NCX expression.^8,9^

The strength of this study is the evaluation of the functional changes in isolated papillary muscles in hyperthyroidism. The major limitation, which is also a suggestion to be explored in other studies, is the lack of molecular biology experiments evaluating the expression of membrane proteins that control intracellular calcium and the calcium content in the SR.

## Conclusions

We demonstrated in this study that isolated papillary muscles from hyperthyroid animals presented decreased PRP, increased +df/dt and -df/dt, low positive inotropic response to decreased concentrations of extracellular sodium, decreased production of maximum force induced by caffeine, and decreased time to reach both the contraction peak and maximum relaxation. We hypothesize that these changes are likely due to a decrease in calcium content in the SR, probably caused by calcium leakage, decreased expression and/or activity of NCX, and increased expression of a-MHC and SERCA2.
